# Corn-Starch-Based Materials Incorporated with Cinnamon Oil Emulsion: Physico-Chemical Characterization and Biological Activity

**DOI:** 10.3390/foods9040475

**Published:** 2020-04-10

**Authors:** Edaena Pamela Díaz-Galindo, Aleksandra Nesic, Silvia Bautista-Baños, Octavio Dublan García, Gustavo Cabrera-Barjas

**Affiliations:** 1 Facultad de Química, Universidad Autónoma del Estado de México, Km 115 Carr. Toluca-Ixtlahuaca. El Cerrillo Piedras Blancas, Toluca 50295, Mexico; pam.dg12@hotmail.com (E.P.D.-G.); odublang@uaemex.mx (O.D.G.); 2 Unidad de Desarrollo Tecnológico, UDT, Universidad de Concepción, Avda. Cordillera No. 2634, Parque Industrial Coronel, Coronel 4191996, Chile; 3 Vinca Institute for Nuclear Sciences, University of Belgrade, Mike Petrovica-Alasa 12–14, Belgrade 11000, Serbia; 4 Instituto Politécnico Nacional, Centro de Desarrollo de Productos Bióticos (CEPROBI), Carretera Yautepec-Jojutla, Km. 6, calle CEPROBI No. 8, Col. San Isidro, Yautepec, Morelos 62731, Mexico; silviabb2008@hotmail.com

**Keywords:** starch films, active food packaging films, cinnamon oil emulsions, *Botrytis cinerea*

## Abstract

Active packaging represents a large and diverse group of materials, with its main role being to prolong the shelf-life of food products. In this work, active biomaterials based on thermoplastic starch-containing cinnamon oil emulsions were prepared by the compression molding technique. The thermal, mechanical, and antifungal properties of obtained materials were evaluated. The results showed that the encapsulation of cinnamon oil emulsions did not influence the thermal stability of materials. Mechanical resistance to break was reduced by 27.4%, while elongation at break was increased by 44.0% by the addition of cinnamon oil emulsion. Moreover, the novel material provided a decrease in the growth rate of *Botrytis cinerea* by 66%, suggesting potential application in food packaging as an active biomaterial layer to hinder further contamination of fruits during the storage and transport period.

## 1. Introduction

In the last decade, the Chilean fruit industry has been consolidated as one of the main international leaders in the export of fresh fruits, particularly grapes, strawberries, and raspberries. Fruit exports accounted for 27% of the sector’s total export value in 2016, with an export value of US$16 billion, which makes this sector the most important in the country, being surpassed only by the mining industry [1]. However, the appearance of gray mold caused by the fungal contamination of fruits poses a big problem, accounting for approximately 20% of fruit losses during storage and transport. *Botrytis cinerea* is the most widespread fungal disease on fruits and is mainly manifested in the post-harvest period.

Recently, active biodegradable packaging has gained more importance for fruit storage directly after harvesting, in order to minimize the appearance of gray mold and losses during transport. Namely, this type of packaging can be directly in contact with the surface of food products or with the headspace between the package and the food products. Moreover, active materials can appear in the form of sachets/capsules that contain an antifungal agent that is inserted into a package or in the form of inner coating of the packaging material. The role of the antifungal agent is to reduce, inhibit, or hinder the growth of fungi that may be present in the packed fruits [2,3,4,5]. Moreover, special attention is paid to the use of bioactive components such as plant-derived essential oils, due to their high antifungal/antimicrobial and antioxidant activity [6,7,8,9,10]. Incorporation of essential oils into polymer package presents a big technological challenge because of their evaporation during the melting processing of the polymer. Such a challenge can be solved by the inclusion of essential oil in the biopolymer matrix and the encapsulation of stable emulsion into a thermoplastic polymer that is processed at a lower temperature than a temperature at which essential oil evaporates/degrades. Moreover, this material could prevent easy penetration of volatiles into the food, protecting the items from coming in contact with substances that could affect their taste and odor.

Among all biopolymers, starch is a very promising candidate for the processing of biodegradable food packaging materials [11,12]. Starch can be found in plants such as corn, wheat, rice, and peas, so its use is expanded due to its low price and easy availability. Starch has thermoplastic behavior in the presence of plasticizers and when elevated temperature and shear are applied. In fact, the processing of thermoplastic starch (TPS) is possible by the use of conventional techniques for synthetic polymers, such as compression-molding, injection molding, and extrusion blow molding [13]. TPS-based materials have been commercialized over the last decade and are currently used in the food sector as single-use packages, for example, egg trays, plates, and cups. One of the greatest benefits of TPS is that it can be processed at significantly lower temperatures (90–140 °C) in comparison to other bioplastics/plastic materials (180–230 °C), allowing safe operation with volatile bioactive components (degradation around 180 °C), thus minimizing the loss during processing.

In this work, cinnamon oil was used as an antifungal component, because has already been proven to be an efficient bioactive agent toward *Botrytis cinerea* [14,15,16]. In order to minimize the losses during processing, stable water in oil emulsion was prepared in the presence of mucilage. Mucilage extracted from chia seeds shows high emulsifying activity and may also act as a stabilizer of emulsions [17,18,19,20,21]. Furthermore, stable emulsions were incorporated into TPS, in order to obtain antifungal biodegradable material that could be used as an inner layer of active packaging. Thermal, mechanical, and antifungal properties toward *Botrytis cinerea* were assessed.

## 2. Materials and Methods

The cinnamon essential oil was supplied by Cedrosa (Estado de México, Mexico). Chia seeds were purchased from the local market in Mexico. Starch from corn was purchased from Buffalo^®^ 034,010 (CornProducts Chile Inducorn S.A., Santiago, Chile). Glycerol was obtained from OCN company (Qindao, China).

### 2.1. Chia Mucilage Extraction

The extraction of mucilage was performed according to the method proposed by Velázquez-Gutiérrez et al. [22]. Namely, 40 g of chia seeds were soaked in 800 mL of Mili-Q water. The pH of the mixture was adjusted to 8 by using 0.1 M NaOH solution. The mixture was stirred for 2 h at a constant temperature of 80 °C. Afterward, the mucilage was separated from the seed, and the filtrate was centrifuged for 8 min at 524× *g*. The supernatant was decanted and analyzed. The extracted mucilage was frozen, and afterward, the sample was dehydrated using a freeze-dryer for 48 h. The dehydrated products were stored in desiccators with P_2_O_5_ in order to prevent any moisture absorption until experiments that required usage of these products were performed.

### 2.2. Oil-in-Water (O/W) Emulsion Preparation

A certain amount of obtained mucilage was dissolved in water at room temperature for 12 h, with continuous stirring, in order to obtain different concentrations of aqueous solution (0.2–1.5 wt%). The emulsions were made by mixing the mucilage solutions as an aqueous phase and the cinnamon essential oil as a lipid phase with a laboratory T-25 digital Ultraturrax at 9600 rpm for 2 min. The concentration of cinnamon oil in water varied from 1 to 5 v/v (1/99; 2/98; 3/97; 4/96 and 5/95 v/v oil/water). The total volume of the aqueous/water phase was 50 mL. In order to check the stability of emulsions, the creaming index was monitored at specific storage time (0, 30, and 60 days). When creaming occurred during storage time at room temperature (25 °C), emulsions were homogenized to re-disperse the cream layer before the analysis. Three samples per each emulsion formulation were tested, and the deviation was less than 3%.

### 2.3. Characterization of Emulsions

#### 2.3.1. Creaming Index

Each emulsion was evaluated to detect visible parameters such as color, creaming, coalescence, and/or separation of phases. After the emulsions were homogenized and centrifuged for 10 min at 524× *g*, the creaming index (CI, %) was checked and calculated according to the following Equation (1):

(1) $$CI\left(\%\right)=\frac{H_{t}}{H_{o}}\times 100$$

where H_o_ is the total height of the emulsion layer in vials and H_t_ is the height of the cream layer. Analyses were performed in triplicate.

#### 2.3.2. Thermal Stability

The thermal stability (TS, %) of emulsions was evaluated by subsequent heating of the emulsion in a water bath at 80 °C for 30 min and subsequent cooling down to room temperature (20 ± 2 °C), followed by centrifugation for 10 min at 524× *g*. The heights of the emulsified layer and cream layer were measured, and the TS was calculated according to the following Equation (2):

(2) $$\;S\left(\%\right)=\frac{H_{o}-H_{t}}{H_{o}}\times 100$$

where H_o_ is the total height of the emulsion layer in vials and H_t_ is the height of the cream layer. Analyses were performed in triplicate.

#### 2.3.3. In Vitro Antifungal Activity of Emulsions

Twenty milliliters of sterilized potato dextrose agar (PDA) was placed in Petri dishes (100 × 15 mm). A volume of 0.1 mL of the emulsion was uniformly dispersed in the culture medium PDA (Bioxon) on six Petri dishes per treatment and allowed to dry. A disc of 5 mm in diameter of *B. cinerea* was placed in the center of the Petri dishes and incubated at 25 ± 2 °C until control (with sterile water) reached its maximum development. The plates were sealed with Parafilm^®^ to avoid vapor leakage.

Mycelial growth over time was measured daily using a Vernier caliper. The test ended when the mycelium completely covered the Petri dish in the control sample. Six repetitions per treatment were carried out. The mycelial growth inhibition index (IM) was calculated according to the following Equation (3):

(3) $$IM\left(\%\right)=\frac{C_{C}-C_{T}}{C_{C}}\times 100$$

where C_C_ is the control’s growth, and C_T_ is the growth in the treatment group. Analyses were carried out in triplicate.

### 2.4. Preparation of Thermo-Plasticized Starch-Emulsion Plates

The first step in the preparation of materials was the thermo-plasticization of starch. The starch was homogenized with 150 g of glycerol and 50 g of water in a high-speed blade mixer (Cool Mixer, Labtech model LCM-24) at 45 °C and a speed of 2800 rpm. This sample was coded as TPS and used to prepare control the TPS plate by a compression molding technique. According to the preliminary results related to emulsions, the two best formulations were chosen to be incorporated into the starch matrix during the thermo-plasticization process. The same procedure was followed to obtain the thermo-plasticized starch loaded with emulsions, as for the control TPS sample.

Plates were made by the use of Labtech LP-20B hydraulic press. The 40 g of thermo-plasticized starch samples was placed between two stainless steel molds that were covered with a Teflon sheet. The samples were compressed with an applied pressure of 70 bar for 3 min at 140 °C. The resulting plates were cooled for 1 min before unmolding. The thickness of the obtained materials was approximately 0.5 mm.

### 2.5. Characterization of Plates

#### 2.5.1. TGA

Thermogravimetric analysis (TGA) was performed by NETZSCH TG 209 F3 Tarsus^®^. The operating conditions were as follows: nitrogen flow of 10 mL/min, temperature heating range from 30 to 500 °C, and a heating rate of 10 C/min. All measurements were performed in triplicate, and obtained parameters were repeatable within ±3%.

#### 2.5.2. Mechanical Analysis

Mechanical analysis was performed on a Universal test machine KARG Industrie Technik Smartens 005) according to the ASTMD638 (2010) standard test method. The test conditions were as follows: 23 ± 2 °C, 45 ± 5% RH, crosshead speed 2 mm/min. The measurements were carried out in sextuplicate. The standard deviation for the tested parameters was ± 10%.

#### 2.5.3. In Vitro Antifungal Activity

The PDA culture medium and cultivation of *B. cinerea* was prepared as described in Section 2.3.3. The antifungal films were cut into 1 cm diameter pieces and attached to the inside cover of the Petri dishes. The Petri dishes were then sealed with Parafilm colony diameters (cm) in each Petri dish within the time they were monitored. As a control sample, starch films without antifungal compounds were used. Analyses were carried out in triplicate.

## 3. Results

### 3.1. Emulsions

The concentration of mucilage was shown to play a significant role in the stabilization of emulsions. In fact, emulsion was only obtained when the used mucilage content was above 0.75 wt%. Table 1 presents the values of the creaming index (CI) and thermal stability of emulsion formulations containing 1 wt% and 1.5 wt% of mucilage at zero-day, after 30 days and 60 days of storage at room temperature. The highest stability of emulsions was obtained when 1.5 wt% of mucilage was used, since creaming did not appear even after 60 days of storage, and the evaluated thermal stability was 99%. Other authors previously reported 120 days stability of w/o emulsion when chia mucilage was added at 0.75 and 1 wt%, respectively [18]. These results are in agreement with those obtained by Guiotto et al. [23] who prepared w/o emulsion and added chia mucilage (0.75 wt%) as a stabilizer. The mucilage addition contributed to the stabilization of the emulsion CI for 120 days. The authors correlated these results in a three-dimensional network, which showed reduced oil droplets mobility inside the emulsion. It has been previously reported that the emulsifying properties of chia mucilage could be associated with a certain protein content in its structure. Such proteins could contribute to the surface activity of chia mucilage dispersions [24].

**Figure 1:**
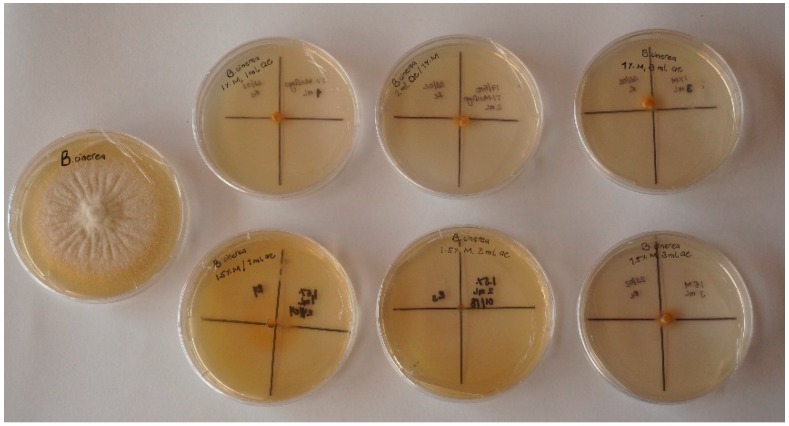
Antifungal activity of cinnamon oil emulsions.

The effects of different emulsion formulations on the radial growth of *B. cinerea* are shown in Figure 1. The highest radial growth was obtained in the control sample (without emulsion application). All tested emulsions (see Table 1) completely inhibited the growth of *B. cinerea*. It is important to highlight that after two months of storage, all tested emulsions were again subjected to antifungal tests and again showed 100% growth inhibition. The high antifungal activity of cinnamon oil is already well known because of its chemical composition [25,26]. Namely, the main constituent of cinnamon oil is cinnamaldehyde, which contains an aldehyde group and a conjugated double bond outside the ring. These groups are responsible for the deactivation of enzymes in fungi [27]. Few studies have shown that cinnamon oil inhibits the biosynthesis of ergosterol, the major sterol constituent of the fungal plasma membrane, which leads to damage of the cell membrane structure, and consequently, the leakage of intracellular ions [28]. Hence, cinnamon oil stabilized by mucilage could be a good bioactive candidate for thermoplastic bio-packages to prevent or hinder the growth of *B. cinerea*. Since the best emulsion stability within the time showed B1i–B3i formulations, these formulations were chosen for further incorporation into thermoplastic starch (Table 2).

### 3.2. Characterization of Starch/Emulsion Materials

#### 3.2.1. Mechanical Analysis

The mechanical parameters, values of the tensile strength (TS), and percentage of elongation at break (e) of the materials are presented in Table 3. The neat thermoplasticized starch film exhibited an average tensile strength value of 2 MPa and an elongation at break value of 50.5%. The incorporation of emulsions into the starch matrix resulted in a decrease in tensile strength when compared with one of the neat starch films. The elongation at break value of films increased with the addition of essential oil emulsion in the starch matrix. As presented in Table 3, a reduction of approximately 24% in TS% value and an increase in plasticity by approximately 70% were obtained by encapsulation of B2i emulsion into starch. The majority of data from the literature provide evidence of a decrease in TS and an increase in elongation at break of films when essential oils are introduced in polysaccharide matrices, such as chitosan [29], starch [30], pectin [31], and alginate [32]. This trend was explained by the specific interactions between phenolic compounds from essential oils and functional groups from the biopolymer matrix that lead to more elastic matrices [33]. In fact, previous studies have reported that essential oils have a plasticizing effect on biopolymers and diminish the strong intermolecular chain–chain interactions in the polymer structure, thus imparting higher flexibility of films up to the break [34,35]. So far, data in the literature related to the incorporation of inclusion complexes into the thermoplastic biopolymer matrix are scarce. As a carrier of bioactive cinnamon oil and D-limonene, β-cyclodextrin was used and further incorporated into PLA [36] and PBS [37], respectively. However, there are no data related to the mechanical properties of these materials. Moreover, to the best of our knowledge, no data in the literature are found regarding the incorporation of emulsions into thermoplastic polymers.

#### 3.2.2. Thermal Analysis

**Figure 2:**
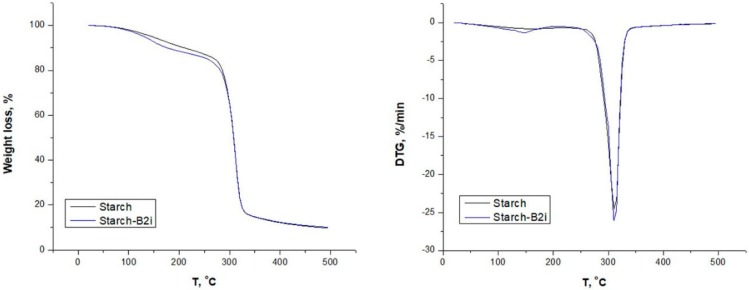
Thermal diagrams of starch and starch-emulsion materials.

The weight loss at 180 °C (W_L180_), the temperature at which degradation starts (T_onset_), the maximum weight loss temperature (T_deg_), and char residue are reported in Table 4. Neat thermoplastic starch displayed two degradation steps (Figure 2). The first degradation step occurred up to 180 °C, where bonded and unbonded water was released, whereas the second step with T_onset_ at 280 °C and maximum degradation peak at 312 °C were related to starch chain decomposition. The addition of emulsion did not affect the thermal degradation profile of thermoplastic starch since there were no significant changes in T_onset_ and T_deg_ values. On the other side, a slight increase in weight loss up to 180 °C and char residue for starch-emulsion plates was observed. These results were expected because low concentrations of volatile components were introduced in thermoplastic starch. The unchanged thermal stability after the inclusion of essential oils/bioactive components were also observed in the literature for LDPE films incorporated with cinnamon and rosemary oil [38] for PLA films containing D-limonene [39] and for PLA films loaded with oregano oil [40].

#### 3.2.3. Antifungal Activity

**Figure 3:**
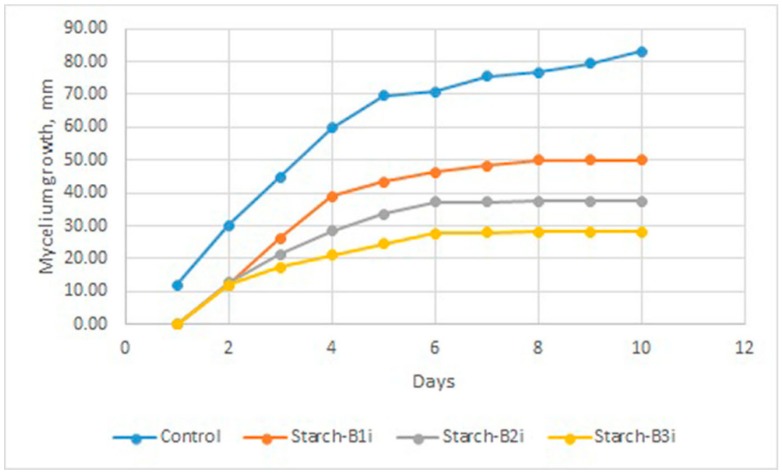
Mycelium growth of *B.cinerea* in the presence of starch and starch-emulsion materials.

The main purpose of the antifungal tests was to evaluate the potential use of starch/emulsion plates as antifungal biodegradable layers/sachets in the food packaging industry, taking into account that *B. cinerea* is well known as a contaminant of fruits and vegetables. Figure 3 displays the fungal growth inhibition within the incubation time of *B. cinerea* at 25 °C. The neat starch plate did not show any antifungal activity, as was expected. Moreover, starch-emulsion plates did not show fungistatic activity but provided a lower rate of mycelium growth. However, it is important to underline that there was limited development of hyphae, and no spore germination was observed, which is important in the prevention of further acceleration of fungi contamination on fruits. The results revealed that mycelium growth inhibition (%) depended on the concentration of bioactive components included in the thermoplastic starch plates. With an increase in the cinnamon oil concentration in thermoplastic starch plates, a lower rate of *B. cinerea* growth was observed. In fact, the inhibition of mycelium growth was above 50% after 10 days of incubation only for samples Starch-B2i and Starch-B3i when compared with the control. This outcome could be explained by a low concentration of cinnamon oil in starch plates, ranging from 0.2 to 0.6 wt%. The inhibition of growth rate of Starch-B3i sample was improved by 66% in comparison with that of the control sample, which means that this material could be used in food packaging as a supporting layer inserted in the box, but only to hinder the further contamination of fruits during storage or transport, minimizing fruit loss and damage. In order to obtain biobased materials with higher antifungal efficiency, further optimization of the system is required. The main optimization of the plasticizer and emulsion ratio with respect to the starch matrix is necessary in order to avoid a further decrease in the mechanical stability of the final materials. In fact, introducing a higher amount of emulsion into the starch matrix would further increase the antifungal activity and elasticity of the material but would significantly reduce its tensile strength, which can be an undesirable effect from the industrial point of view. Moreover, a higher concentration of emulsion could cause olfactory and gustatory contamination of packed foods (off-flavor, off-odor) due to the migration of volatile compounds from package to food. Hence, besides mechanical and biological stability, further studies should include the evaluation of the side effects of materials containing cinnamon oil emulsion on the sensory properties of food (odor and flavor).

## 4. Conclusions

The study investigated the potential use of thermoplastic starch incorporated with cinnamon oil emulsion as a bioactive antifungal material in the food packaging industry. The addition of cinnamon oil emulsion did not affect the thermal stability of thermoplastic starch. In contrast, the mechanical properties showed a clear enhancement in elongation of obtained bioactive material at the break point. Moreover, the highest loading of the emulsion into thermoplastic starch showed inhibition of the growth of *B.* *cinerea* in the “in vitro” antifungal test. These results demonstrate that thermoplastic starch loaded with cinnamon oil emulsion could be potentially used as a bioactive layer or emitter in the food packaging sector to hinder further infection of fruits.

## Figures and Tables

**Table 1:** Stability of emulsions in different interval period.

Sample Code	Mucilage (wt %)	Cinnamon Oil (mL)	CI %			TS %		
			**0.d**	**30.d**	**60.d**	**0.d**	**30.d**	**60.d**
**A1i**	1	1	0	0	1	100	100	99
**A2i**	1	2	0	0	1	100	98	97
**A3i**	1	3	0	0	1	95	97	95
**B1i**	1.5	1	0	0	0	100	100	99
**B2i**	1.5	2	0	0	0	100	100	99
**B3i**	1.5	3	0	0	0	100	100	99

**Table 2:** Compositions and codes of bioactive biodegradable plates.

Sample Code	Starch (kg)	Glycerol (g)	Water (g)	Emulsion (g)
**Starch**	0.5	150	50	0
**Starch-B1i**	0.5	150	0	50
**Starch-B2i**	0.5	150	0	50
**Starch-B3i**	0.5	150	0	50

**Table 3:** Mechanical parameters of starch-based plates.

Sample Code	TS (MPa)	e (%)
**Starch**	2.04	50.5
**Starch-B1i**	1.65	84.4
**Starch-B2i**	1.55	86.2
**Starch-B3i**	1.48	90.3

**Table 4:** TGA parameters for starch-based plates.

Simple	W_L180_ (%)	T_onset_ (°C)	T_deg_ (°C)	Char Residue at 500 °C (%)
**Starch**	7.8	280	312	9.8
**Starch-B1i**	11	279	312	10.9
**Starch-B2i**	10.3	278	312	11.0
**Starch-B3i**	9.1	278	312	11.2
